# Diagnostic Methods and Risk Factors for Severe Disease and Mortality in Blastomycosis: A Retrospective Cohort Study

**DOI:** 10.3390/jof7110888

**Published:** 2021-10-20

**Authors:** Timothy R. O’Dowd, Jack W. Mc Hugh, Elitza S. Theel, Nancy L. Wengenack, John C. O’Horo, Mark J. Enzler, Paschalis Vergidis

**Affiliations:** 1 Department of Medicine, Mayo Clinic, Rochester, MN 55905, USA; todowd@bwh.harvard.edu (T.R.O.); mchugh.jack@mayo.edu (J.W.M.H.); 2 Department of Laboratory Medicine and Pathology, Division of Clinical Microbiology, Mayo Clinic, Rochester, MN 55905, USA; theel.elitza@mayo.edu (E.S.T.); wengenack.nancy@mayo.edu (N.L.W.); 3 Department of Medicine, Division of Infectious Diseases, Mayo Clinic, 200 First St. SW, Rochester, MN 55905, USA; ohoro.john@mayo.edu (J.C.O.); enzler.mark@mayo.edu (M.J.E.); 4 Department of Medicine, Division of Pulmonary and Critical Care Medicine, Mayo Clinic, Rochester, MN 55905, USA

**Keywords:** blastomycosis, serology, immunodiffusion, complement fixation, urine antigen, PCR, lymphopenia, amphotericin B, itraconazole

## Abstract

**Background:** Blastomycosis can cause severe disease with progressive respiratory failure and dissemination even in immunocompetent individuals. We sought to evaluate risk factors for severe disease and mortality using clinical and laboratory data within a large health system in an endemic area. **Methods:** We performed a retrospective cohort study of patients diagnosed with blastomycosis at all Mayo Clinic sites from 1 January 2004 through 31 March 2020. Diagnosis was established by culture, histopathology/cytopathology, serology, antigen testing, or PCR. Disease was categorized as mild for patients treated in the outpatient setting, moderate for hospitalized patients who did not require intensive care, and severe for patients admitted to the intensive care unit. Logistic regression was used to evaluate risk factors for severe disease. A Cox proportional hazards model was constructed to evaluate mortality. **Findings:** We identified 210 patients diagnosed with blastomycosis. Mean age was 51 years (range, 6–84). Most subjects were male (71.0%). Extrapulmonary disease was confirmed in 24.8%. In this cohort, 40.5% of patients had mild disease, 37.6% had moderate disease, and 21.9% had severe disease. Independent risk factors for severe disease were neutrophilia (odds ratio (OR) 3.35 (95% CI 1.53–7.35), *p* = 0.002) and lymphopenia (OR 3.34 (95% CI 1.59–7.03), *p* = 0.001). Mortality at 90 days was 11.9%. Median time from diagnosis to death was 23 days (interquartile range 8–31 days). Independent risk factors for mortality were age (OR 1.04 (95% CI 1.01–1.08), *p* = 0.009), neutrophilia (OR 2.84 (95% CI 1.04–7.76), *p* = 0.041), and lymphopenia (OR 4.50 (95% CI 1.67–12.11), *p* = 0.003). *Blastomyces* immunodiffusion had an overall sensitivity of 39.6% (95% CI 30.1–49.8). Sensitivity was higher among those who were tested 4 weeks or longer after the onset of symptoms. Urine *Blastomyces* antigen had a significantly higher sensitivity of 80.8% (95% CI 68.1–89.2) compared to serology. There was a trend towards higher antigen concentration in patients with severe disease. The sensitivity of PCR from respiratory specimens was 67.6% (95% CI 50.1–85.5). **Conclusion:** In this cohort, we did not find an association between pharmacologic immunosuppression and disease severity. Lymphopenia at diagnosis was an independent risk factor for mortality. This simple marker may aid clinicians in determining disease prognosis.

## 1. Introduction

*Blastomyces dermatitidis* and the more recently described *Blastomyces gilchristii* [1] are thermally dimorphic fungi endemic to the Mississippi, Ohio, and St. Lawrence River basins and the Great Lakes region of the USA and Canada. Other species have also been reported as occasional causes of human disease [2,3]. Infection is primarily acquired by inhalation of conidia and may manifest as acute or chronic pneumonia. Pulmonary infection can progress to respiratory failure and acute respiratory distress syndrome (ARDS). Hematogenous dissemination can occur with involvement of the skin, bone, central nervous system (CNS) or other organs. Disease outbreaks have been reported [4,5]. However, most cases occur sporadically.

The diagnosis is often established by culture and/or histopathology. Serologic assays and antigen testing offer additional diagnostic tools. While not standardized, molecular identification via polymerase chain reaction (PCR) is shown to be a sensitive method for the detection of *Blastomyces* species. Treatment is determined by disease severity. Amphotericin B is reserved for cases of severe pneumonia or disseminated disease, particularly in the immunocompromised host. Itraconazole is the mainstay of treatment for mild-to-moderate disease and is also used as step-down therapy after induction with amphotericin B. Voriconazole and the novel triazoles posaconazole and isavuconazole demonstrate in vitro activity. However, clinical experience is limited, and these agents have not been studied in clinical trials for the treatment of blastomycosis.

The diagnosis may be delayed as patients commonly present with symptoms suggestive of bacterial pneumonia and receive empiric antibacterial treatment [6,7]. Skin manifestations may provide an early clue to diagnosis [8]. An increase in the incidence of blastomycosis has been reported in recent years possibly related to climate changes [9,10]. Herein, we examine risk factors for severe disease and mortality using clinical and laboratory data within a large health system in an endemic area for blastomycosis. We also evaluate the sensitivity of the currently used diagnostic methods.

## 2. Methods

### 2.1. Study Cohort

We performed a retrospective cohort study of patients diagnosed with blastomycosis at all Mayo Clinic sites from 1 January 2004 through 31 March 2020. The Upper Midwest Mayo Clinic Health System is comprised of a tertiary referral hospital in Rochester, Minnesota, and a network of regional hospitals in southern Minnesota and western Wisconsin; areas located in the Mississippi River basin. The study was approved by the Institutional Review Board. Subjects were identified by review of ICD-9/ICD-10 diagnostic codes and microbiology/serology/pathology data. Disease was defined in patients with clinical and/or radiographic evidence of infection and one or more of the following: (i) isolation of *Blastomyces* in culture of respiratory, skin or sterile specimens, (ii) histopathologic or cytopathologic evidence of yeast consistent with *Blastomyces*, (iii) positive serology (immunodiffusion or complement fixation titer ≥ 1:16), (iv) positive *Blastomyces* antigen in urine or serum, or (v) positive *Blastomyces* PCR. 

Disease was categorized as mild for patients treated in the outpatient setting, moderate for hospitalized patients who did not require intensive care, and severe for patients admitted to the intensive care unit (ICU). 

The following variables were studied: age, sex, race/ethnicity, asthma/chronic obstructive lung disease (COPD), diabetes mellitus (treated with oral antidiabetic agents or insulin), renal failure (defined as creatinine ≥ 2.0 mg/dL or eGFR < 30 mL/min/1.73 m^2^), obesity (body mass index ≥ 30 kg/m^2^), corticosteroid treatment (defined as use of prednisone ≥ 20 mg/day or equivalent corticosteroid dose for >4 weeks), other pharmacologic immunosuppression and presence of solid organ transplant. Neutrophilia was defined as neutrophil count ≥ 7.5 × 10^3^ cells/microL and lymphopenia as lymphocyte count < 1.0 × 10^3^ cells/microL. Laboratory values were abstracted at diagnosis of blastomycosis. When available, the mean of 2 values was used for differential cell count. Patients were followed up as needed for their medical care. 

### 2.2. Diagnostic Assays

Specimens were cultured on selective fungal media (e.g., inhibitory mold agar and brain heart infusion blood agar with chloramphenicol and gentamicin with the addition of cycloheximide for respiratory specimens). Cultures were incubated for 24 days at 30 °C. Preliminary identification of fungi was based on colonial and microscopic morphology. Results were reported as *Blastomyces dermatitidis*/*gilchristii* as the two species are morphologically indistinct. Culture isolates were identified using matrix-assisted laser desorption ionization-time of flight (MALDI-TOF) mass spectrometry and/or D2 ribosomal RNA (rRNA) gene sequencing. 

Since 2010 the OMEGA *Blastomyces* Total Antibody enzyme immunoassay (EIA) was used as a screening serologic test (IMMY, Norton, OK, USA) [11]. For positive EIA, confirmatory testing by immunodiffusion (IF) was performed. Before 2010, antibodies to the mycelial and yeast forms of *Blastomyces dermatitidis* were also detected by complement fixation (CF). The MVista^®^ Quantitative Sandwich Enzyme Immunoassay was used for antigen testing (MiraVista Diagnostics, Indianapolis, IN, USA). Results below 0.2 ng/mL or above 14.7 ng/mL fall outside the linear range of the assay and were reported as positive below or above the limit of quantification, respectively. For the laboratory-developed real-time *Blastomyces* PCR assay, primers and FRET hybridization probes were designed to target a 174-base pair region of the histidine kinase (*DRK-1*) gene [12]. We assessed cross-reactivity with other non-culture-based assays. Urine *Histoplasma* antigen testing was performed using the IMMY galactomannan enzyme immunoassay. For serum and BAL (bronchoalveolar lavage) *Aspergillus* galactomannan we used the Platelia™ *Aspergillus* Ag (Bio-Rad Laboratories, Hercules, CA, USA). (1,3)-β-D-glucan testing was performed using the Fungitell^®^ assay (Associates of Cape Cod, East Falmouth, MA, USA). 

### 2.3. Statistical Methods

Descriptive statistics were used to summarize the cohort. Categorical variables were compared using the χ^2^ or Fisher exact test, as appropriate. Continuous variables were compared using one-way ANOVA. We calculated the confidence interval of the sensitivity of diagnostic assays using the Wilson procedure without correction for continuity. *Blastomyces* antigen concentrations below the level of quantification were assigned a value of 0.2. Concentrations above the level of quantification were assigned a value of 14.7. Univariate logistic regression was used to evaluate risk factors for severe disease. Odds ratios with 95% confidence intervals were determined for each risk factor. Factors found to be associated with severe disease at the 0.10 significance level in univariate analysis were included in the multivariate model. We assessed 90-day mortality. Day 0 was considered the day that antifungal treatment was initiated (for patients who did not receive antifungal treatment, or their treatment was unknown, day 0 was considered the day of diagnosis). A multivariable Cox proportional hazards model was constructed to evaluate mortality. Variables with *p*-values < 0.10 were included in the multivariable model. Survival functions were estimated using the Kaplan–Meier method. The log-rank test was used to compare survival distributions. All comparisons were considered significant for *p*-value < 0.05 (two-tailed test). Statistical analysis was performed using STATA, version 16.1 (StataCorp, College Station, TX, USA) and GraphPad Prism, version 8.0 (GraphPad Software, San Diego, CA, USA).

## 3. Results

We identified 210 patients diagnosed with blastomycosis during the study period (Table 1). Diagnosis was based on culture in 140 subjects, pathology in 46, *Blastomyces* antigen in 11, *Blastomyces* serology in 11, and PCR in 2. Cases by year of diagnosis are shown in Supplementary Table S1. Mean age at diagnosis was 51 years (range, 6–84). Most were male (71.0%, 149/210). In our cohort, 21.9% (46/210) were receiving pharmacologic immunosuppression. None were diagnosed with HIV infection. The diagnosis of blastomycosis was established by culture of respiratory specimens in 61.9% (130/210). Extrapulmonary disease was diagnosed by culture or histopathology in 24.8% (52/210). Of those, 19 had disease limited to the skin or subcutaneous tissue, all of whom were treated as outpatients. CNS involvement was diagnosed in 2.9% (CSF culture and histopathology (1), histopathology alone (1), CSF serology (1), CSF *Blastomyces* antigen (1), MRI abnormalities (2)). Histopathology showed granulomatous inflammation in 56.7% (68/120) and presence of yeast in 65.8% (79/120) of biopsied specimens. In terms of radiographic findings, pulmonary nodule or mass was found in 69.4% (120/173), consolidation in 46.8% (81/173) and cavity in 21.4% (37/173).

**Figure 1:**
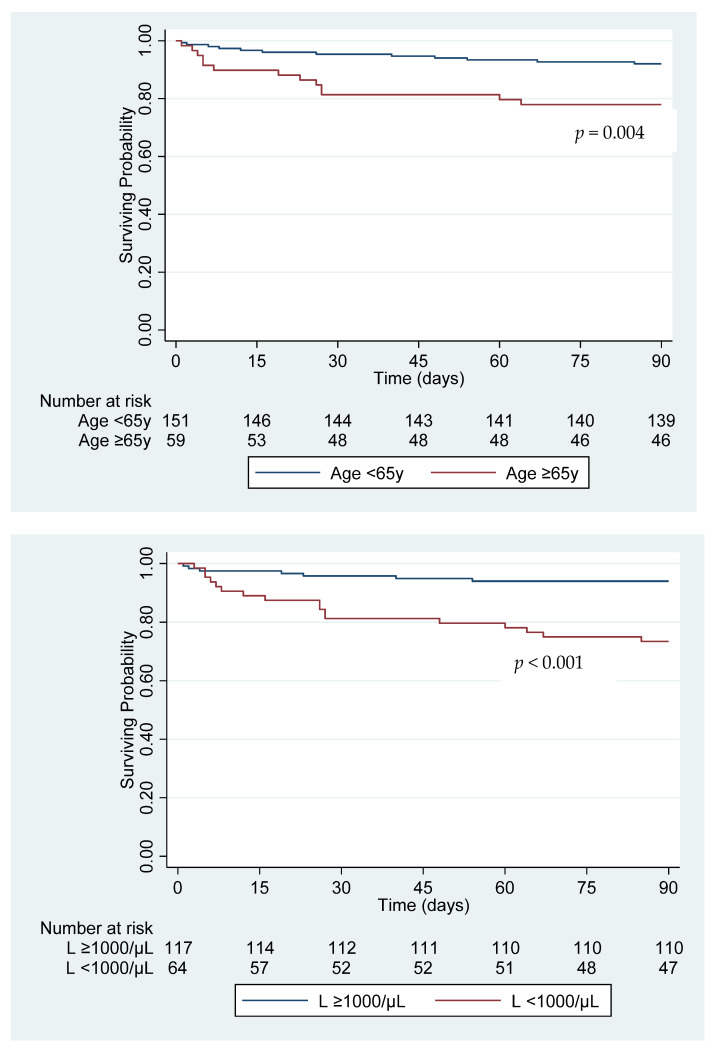
Cumulative 90-day survival grouped by age and presence of lymphopenia at diagnosis. L: lymphocyte count.

In this cohort, 40.5% (85/210) of patients were treated as outpatients (mild disease), 37.6% (79/210) were admitted to the hospital but did not receive intensive care (moderate disease), and 21.9% (46/210) were admitted to the ICU (severe disease). Mechanical ventilation was required for 14.8% (31/210) and extracorporeal membrane oxygenation support for 1.4% (3/210). Older age, COPD, diabetes mellitus, obesity, extrapulmonary disease and immunosuppressive therapy were not associated with the need for ICU admission. Independent risk factors for severe disease were lymphopenia and neutrophilia at the time of diagnosis (Table 2). Mortality at 90 days was 11.9% (25/210) (2.4% (2/85) for mild disease, 5.1% (4/79) for moderate disease, 41.3% (19/46) for severe disease). Median time from diagnosis to death was 23 days (IQR (interquartile range), 8–31 days). The main cause of death was multiorgan failure. Independent risk factors for mortality were age, neutrophilia, and lymphopenia (Table 3). Survival curves grouped by age and lymphopenia are shown in Figure 1. 

Diagnostic assays performed on respiratory specimens are shown in Table 4. KOH/calcofluor white smear was positive in 46.7% (42/90). Sensitivity was higher in BAL/bronchial washings compared to sputum, although this difference did not reach statistical significance. BAL fluid analysis was performed in 33 patients. Medial count of total nucleated cells was 77/microL (IQR, 19–409). Neutrophils were the predominant cells in 57.6% (19/33) and alveolar macrophages in 42.4% (14/33). The sensitivity of PCR from respiratory specimens was 67.6% (45.5% for sputum, 76.9% for lower respiratory tract specimens). CSF PCR was negative in all three specimens of patients with CNS blastomycosis.

Findings of serologic and antigen testing are shown in Table 5. *Blastomyces* immunodiffusion had a sensitivity of 39.6% and complement fixation of 22.9%. There was no difference in sensitivity between pulmonary and extrapulmonary disease. Sensitivity of immunodiffusion was higher among those who were tested 4 weeks or longer after the onset of symptoms compared to those who were tested earlier (51.9% (28/54) vs. 24.0% (6/25), *p* = 0.03). Sensitivity was similar between immunocompetent and immunosuppressed individuals (40.3% (29/72) vs. 36.8% (7/19), *p* = 1.00), even after excluding patients who were diagnosed in less than 4 weeks after the onset of symptoms.

**Figure 2:**
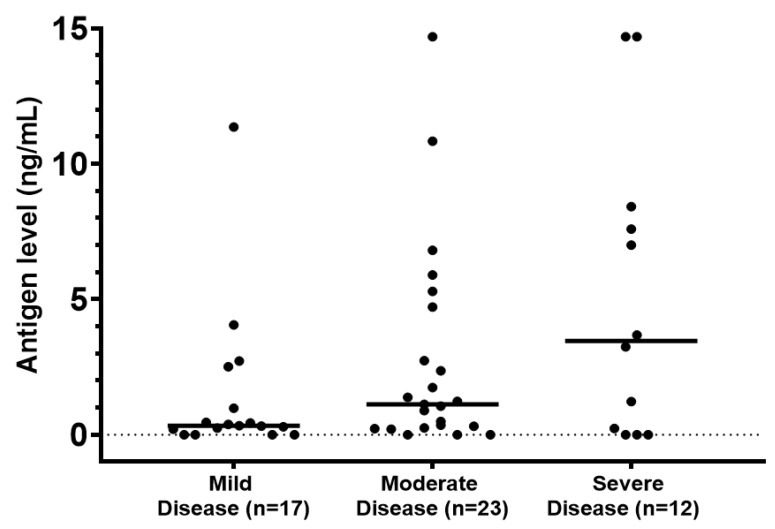
Disease severity and *Blastomyces* urine antigen level. Horizontal lines represent mean values (*p* = 0.06).

Urine *Blastomyces* antigen had a significantly higher sensitivity of 80.8% compared to serology. There was a trend towards higher antigen concentration in patients with severe disease (Figure 2). Urine antigen sensitivity was higher in patients with pulmonary disease compared to those with extrapulmonary infection confirmed by culture and/or histopathology. In patients with positive *Blastomyces* serology we noted cross-reactivity with the *Histoplasma* complement fixation assay. However, none of the seven tested patients had positive *Histoplasma* immunodiffusion. There was also cross-reactivity with urine *Histoplasma* antigen. Of note, BAL *Aspergillus* galactomannan was positive in 7 of 19 patients with culture-proven blastomycosis. Serum *Aspergillus* galactomannan was negative in all 36 tested patients. (1,3)-β-D-glucan was positive in 1 of 12 patients at a value of 252 pg/mL without evidence of other concomitant fungal infection.

In terms of antifungal therapy, 38.9% (81/208) of patients received induction treatment with a formulation of amphotericin B. Of patients treated with an azole (with or without induction with amphotericin B), 90.7% (166/183) received itraconazole and 9.3% (17/183) voriconazole as the first azole. As shown in Supplementary Table S2, voriconazole was more commonly used as the first azole in patients with CNS involvement. All other clinical characteristics were similar between the two groups. 

Three patients (1.4%) had disease relapse/recurrence. Their clinical characteristics are summarized in Table 6. None of the patients had a known immunocompromising condition. Based on clinical review, patient 1 probably had re-exposure to *Blastomyces* conidia that led to the recurrence with pulmonary consolidation on the contralateral lung. Patient 2 had chronic cavitary blastomycosis. After the first disease relapse, she had persistent low-positive antigen concentrations, and eventually developed a new masslike consolidation in the inferior margins of the cavity. Patient 3 appeared to have partial response to initial treatment with itraconazole (drug concentration was not measured) and was retreated with posaconazole after he was found to have new bone abnormalities.

## 4. Discussion

We reviewed clinical characteristics, diagnostic methods and outcomes in patients diagnosed with blastomycosis over a 16-year period. Our cohort includes 210 patients of whom 20.5% had an underlying immunocompromising condition and 24.8% had evidence of extrapulmonary dissemination. We found that neutrophilia and lymphopenia at the time of diagnosis were independent risk factors for severe disease. Pharmacologic immunosuppression was not found to be associated with the severity of illness. In a recent single center retrospective study conducted in Wisconsin, immunosuppression was associated with severe disease and increased mortality [13], a finding that was not replicated in our cohort. Both studies covered approximately the same period and noted similar rates of dissemination between immunocompetent and immunocompromised individuals. In another retrospective study conducted in Indiana, diabetes mellitus and immunosuppression were found to be associated with ICU admission [14], which is also in contrast to our findings. We note that in the latter study high-dose corticosteroid use was defined as prednisone ≥ 10 mg/day for >2 weeks, whereas in our study we considered immunosuppressed those receiving ≥ 20 mg/day for >4 weeks. As blastomycosis is a rare condition limited to certain geographic areas, it is difficult to make definitive conclusions on the impact of immunosuppression or other comorbidities on disease outcomes. Microsatellite typing of *B. dermatitidis* isolates suggested that clinical characteristics may be associated with genetic variation of the organism [15]. Blastomycosis can cause severe disease with progression to ARDS in previously healthy individuals. 

In our cohort, 90-day all-cause mortality was 11.9%. We identified increasing age as an independent risk factor for mortality, which is consistent with previous nationwide studies [16,17]. In a large analysis of 1216 blastomycosis-related deaths, using data from the National Center for Health Statistics, mortality was significantly higher in men [16]. These data were derived from US death certificates and included information coded by the International Classification of Diseases. However, in an analysis of 1848 patients with blastomycosis from the National Inpatient Sample, an all-payer database that captures approximately 20% of US hospitalizations, female gender was independently associated with increased mortality in those who required mechanical ventilation [17], a finding not observed in other studies. We also did not find an association between sex and increased mortality. 

Cell-mediated immunity is considered the major host defense against dimorphic fungi. In our study, we had access to detailed laboratory data for most patients. We identified lymphopenia as a risk factor for severe disease and mortality, even after accounting for the effect of immunosuppressive treatment (corticosteroids or other). None of the patients in our cohort were known to be infected with HIV. T-lymphocyte subsets were not obtained thus not allowing for further characterization of the immune profile. A decrease in T-helper lymphocytes was previously reported in patients with untreated pulmonary blastomycosis and an increase in T-helper cell count following antifungal therapy corresponded with clinical improvement [18]. More recent research aimed at fungal vaccine development has demonstrated the protective effect of Interleukin-17A and T-helper type 17 cells against *Blastomyces* [19]. Based on our findings, lymphopenia at presentation is a simple marker that can aid in disease prognostication.

Most patients were expectedly residing in Minnesota and Wisconsin given the geographic location of the Mayo Clinic Health System in the upper Midwest. We noted an increase in the number of cases diagnosed at our institution between 2017 and 2019. We did not perform a population-based study and thus conclusions on the incidence of the disease cannot be drawn. However, the upwards trend in the recent years is consistent with the epidemiologic findings throughout Minnesota which prompted a public health advisory in 2019 [20]. As in previous studies [7,9], we report that the disease is more common in men. This has been attributed to exposure during outdoor activities. In our study, the disease was most commonly diagnosed in white individuals. In Minnesota, higher incidence has been reported among American Indian/Alaska natives (2.7/100,000 population) [7]. Studies in other geographic locations have shown a higher incidence among black patients [21], Aboriginal Canadian [22] and the Hmong population in Wisconsin, possibly related to genetic predisposition [23]. The small proportion of non-white patients in our study precluded an analysis of disease severity and mortality in other racial groups.

Standard of care for patients with severe disease consists of a lipid formulation of amphotericin B followed by step-down therapy to a triazole. As patients may be intolerant to itraconazole, there has been an increased interest in the use of novel triazoles for the management of endemic mycoses. In a single-center retrospective cohort study, use of voriconazole as initial or step-down treatment for histoplasmosis was associated with higher 42-day mortality compared to itraconazole [24]. In our cohort, patients with CNS disease were more likely to be treated with voriconazole given its better penetration into brain tissue [25]. Thus, we decided not to compare outcomes based on the selected azole therapy since CNS disease is associated with higher mortality. In our review we describe causes of disease recurrence/relapse. Individuals residing in endemic areas may get re-exposed. Chronic cavitary disease, which has mainly been described with aspergillosis and histoplasmosis, can also occur in blastomycosis and disease may recur after discontinuation of azole therapy. Finally, dissemination can occur in the setting of partially treated pulmonary disease.

Urine *Blastomyces* antigen was the most sensitive diagnostic assay consistent with findings from previous studies [26,27]. Its performance was similar in patients with pulmonary and disseminated disease. In addition, our findings suggest that antigen level correlates with disease severity, thus providing a prognostic tool to clinicians. Serial measurements of urine *Blastomyces* antigen has been found useful in monitoring response to therapy [28]. The serum *Blastomyces* antigen assay was less sensitive. Using the largest number of serum specimens reported thus far, we provide an update on the utility of serologic testing [29,30]. We found that *Blastomyces* EIA, immunodiffusion, and complement fixation have a low sensitivity. It has been concluded elsewhere that ordering urine *Blastomyces* antigen for suspected disease without serology testing is associated with lower healthcare expenses without compromising sensitivity [31]. We found a high sensitivity of PCR on respiratory specimens. We note that the PCR assay was developed in-house and is not available in other laboratories. Our study was not designed to evaluate the specificity of the diagnostic assays.

*Blastomyces* antigen cross-reacts with assays for histoplasmosis or other endemic mycoses [32]. We found a higher sensitivity of the urine *Blastomyces* antigen compared to urine *Histoplasma* antigen for the diagnosis of culture-/histopathology-confirmed blastomycosis. The difference did not reach statistical significance yet demonstrates the value of dual testing in endemic areas. In terms of serologic assays, we noted cross-reactivity with *Histoplasma* complement fixation but not immunodiffusion. This finding can assist in distinguishing between the two entities when culture results are not available. We previously reported positive BAL *Aspergillus* galactomannan in patients with histoplasmosis [33]. In the current study, we demonstrate BAL galactomannan positivity in a percentage of patients with blastomycosis. Clinicians should be aware of the cross-reactivity, particularly when evaluating immunocompromised hosts at risk for invasive pulmonary aspergillosis. 

Our study has several strengths. The Upper Midwest Mayo Clinic Health System covers an area that is highly endemic for the disease, and this is one of the largest cohorts of patients with blastomycosis from a single institution. Our analysis is based on detailed clinical, laboratory and microbiology data. No patients were lost to follow-up by 90 days. Due to its retrospective design the study has inherent limitations. Practices over the 16-year period of the study have changed (particularly in the ICU setting) and this may have had an impact on outcomes. For some patients the results of certain diagnostic assays (such as differential cell count, serology or *Blastomyces* antigen) were not available. The diagnosis of blastomycosis may have been delayed, and subsequently the laboratory results and imaging studies were obtained at varying stages in the course of the disease. Finally, our findings may not be generalizable to non-white individuals. 

Our findings provide further insight into the epidemiology and outcomes of blastomycosis and set the framework for future research. Patients belonging to different racial or ethnic groups should be further studied to determine potential disparities in the outcomes of blastomycosis. Studies in specific immunocompromised populations may elucidate characteristics in relation to the net state of immunosuppression. The finding of lymphopenia as a marker of disease severity can be further explored to characterize immunologic deficits contributing to increased mortality in blastomycosis. Our study demonstrated the poor sensitivity of the currently available serologic assays. Novel methods such as enzyme immunoassay detection of antibodies against the *B. dermatitidis* surface protein *BAD-1* (not used in our study) may improve diagnostics [34]. 

In this work, we reviewed cases of blastomycosis at a large health system in an area of endemicity. Unlike previous studies, we did not find an association between immunosuppressive therapy or other comorbidities and disease severity. We confirmed the higher sensitivity of *Blastomyces* antigen and PCR testing compared to other diagnostic assays. Older age, neutrophilia and lymphopenia at diagnosis were independent risk factors for mortality and these markers may aid clinicians in determining disease prognosis.

## Figures and Tables

**Table 1:** Epidemiologic and clinical characteristics of the cohort (n = 210).

Characteristic	% (N)
**Male Sex**	71.9 (149/210)
**Race/Ethnicity**	
White	82.4 (173/210)
Black	3.8 (8/210)
Asian	3.8 (8/210)
American Indian	1.4 (3/210)
Hispanic	4.3 (9/210)
Unknown	8.6 (18/210)
**State of Residence**	
Minnesota	43.3 (91/210)
Wisconsin	17.6 (37/210)
Iowa	13.3 (28/210)
Illinois	4.3 (9/210)
Michigan	3.8 (8/210)
North Dakota	3.8 (8/210)
Other	13.8 (29/210)
**Comorbidities**	
Asthma/COPD	9.0 (19/210)
Diabetes mellitus	22.9 (48/210)
Renal failure ^1^	7.3 (14/193)
Obesity ^2^	33.9 (65/192)
Corticosteroid treatment ^3^	7.1 (15/210)
Pharmacologic immunosuppression ^4^	18.6 (39/210)
Solid organ transplant	8.1 (17/210)
**Extrapulmonary Disease**	24.8 (52/210)
**Diagnosis Confirmed by Culture**	
Respiratory secretions/Lung tissue	60.0 (126/210)
Skin/soft tissue	11.0 (23/210)
Paranasal sinus/Larynx	1.4 (3/210)
Bone/Synovial Fluid	1.4 (3/210)
Central Nervous System	0.5 (1/210)
Bone Marrow	0.5 (1/210)
Blood	0.5 (1/210)
Urine	0.5 (1/210)
**Diagnosis confirmed by Histopathology**	
Lung	62.0 (75/121)
Skin/soft tissue	24.8 (30/121)
Mediastinal lymph node	6.6 (8/121)
Pleura	0.8 (1/121)
Vocal cord	1.7 (2/121)
Mastoid	0.8 (1/121)
Bone	1.7 (2/121)
Brain	1.7 (2/121)
Spleen	0.8 (1/121)
**Histopathologic Findings**	
Granulomatous Inflammation	56.7 (68/120)
Presence of Yeast	37.1 (78/120)
**Pulmonary Radiographic Findings**	
Consolidation	46.8 (81/173)
Nodule/Mass	69.4 (120/173)
Cavity	21.4 (37/173)
Antifungal Treatment *	
Liposomal Amphotericin B	38.9 (81/208)
Itraconazole	81.3 (169/208)
Voriconazole	14.4 (30/208)
Fluconazole	2.8 (7/208)
Posaconazole	2.8 (6/208)
Isavuconazole	0.5 (1/208)
No treatment	2.8 (6/208)

^1^ Renal failure: creatinine ≥ 2.0 mg/dL or eGFR < 30 mL/min/1.73 m^2^. ^2^ Obesity (BMI ≥ 30 kg/m^2^). ^3^ Prednisone ≥ 20 mg/day (or equivalent corticosteroid dose) for ≥4 weeks. ^4^ Includes corticosteroid treatment. * Treatment not known in 2 patients.

**Table 2:** Risk factors for severe disease.

	Mild/Moderate Disease (*n* = 164)	Severe Disease (*n* = 46)	Univariate Odds Ratio (95% CI)	*p*-Value	Multivariate Odds Ratio (95% CI)	*p*-Value
**Age in years** **Mean (SD)**	51.2 (18.13)	51.3 (18.81)	1.00 (0.98–1.02)	0.960		
**Male Sex**	70.1 (115/164)	73.9 (34/46)	1.21 (0.58–2.53)	0.617		
**Race**						
White	91.3 (136/149)	86.1 (37/43)	Reference			
Black	3.4 (5/149)	7.0 (3/43)	2.21 (0.50–9.66)	0.294		
Asian	4.7 (7/149)	2.3 (1/43)	0.53 (0.06–4.40)	0.553		
American Indian	0.7 (1/149)	4.7 (2/43)	7.35 (0.65–83.32)	0.107		
Hispanic	3.7 (5/135)	0	-			
**Asthma/COPD**	9.8 (16/164)	6.5 (3/46)	0.65 (0.18–2.32)	0.502		
**Diabetes mellitus**						
All	23.2 (38/164)	21.7 (10/46)	0.92 (0.42–2.03)	0.838		
Insulin-Dependent	7.9 (13/164)	13.0 (6/46)	1.74 (0.62–4.87)	0.290		
**Renal Failure**	5.4 (8/149)	15.6 (7/45)	3.25 (1.11–9.52)	0.032	2.43 (0.79–7.46)	0.119
**Obesity**	31.5 (47/149)	41.9 (18/43)	1.56 (0.78–3.14)	0.210		
**Extrapulmonary Disease**	26.2 (43/164)	19.6 (9/46)	0.68 (0.31–1.53)	0.357		
**Corticosteroid Treatment**	7.9 (13/164)	4.4 (2/46)	0.53 (0.11–2.43)	0.412		
**Pharmacologic Immunosuppression**	19.5 (32/164)	15.2 (7/46)	0.74 (0.30–1.81)	0.509		
**Solid Organ Transplant**	6.7 (11/164)	10.9 (5/46)	1.70 (0.56–5.16)	0.352		
**Neutrophilia** ^1^	43.1 (59/137)	72.7 (32/44)	3.52 (1.67–7.42)	0.001	3.35 (1.53–7.35)	**0.002**
**Lymphopenia** ^2^	27.7 (38/137)	59.1 (26/44)	3.76 (1.85–7.64)	<0.001	3.34 (1.59–7.03)	**0.001**

Data are presented as percentages (absolute numbers shown in parentheses), unless otherwise indicated. CI, confidence interval; COPD, chronic obstructive pulmonary disease. ^1^ Neutrophil count ≥ 7.5 × 10^3^cells/microL. ^2^ Lymphocyte count < 1.0 × 10^3^ cells/microL.

**Table 3:** Risk factors for 90-day mortality.

	Survivors (*n* = 185)	Non-Survivors (*n* = 25)	Univariate Hazards Ratio (95% CI)	*p*-Value	Multivariate Hazards Ratio (95% CI)	*p*-Value
**Age in years** **mean (SD)**	49.9 (1.33)	60.8 (3.37)	1.04 (1.01–1.07)	0.007	1.04 (1.01–1.08)	**0.009**
**Male Sex**	71.4 (132/185)	68.0 (17/25)	0.85 (0.35–2.10)	0.729		
**Race/Ethnicity**						
White	90.6 (154/170)	86.4 (19/22)	Reference			
Black	3.5 (6/170)	9.1 (2/22)	2.71 (0.63–11.62)	0.181		
Asian	4.7 (8/170)	0	-			
American Indian	1.2 (2/170)	4.6 (1/22)	3.19 (0.43–23.83)	0.259		
Hispanic	2.6 (4/156)	5.9 (1/17)	2.26 (0.29–16.53)	0.447		
**Asthma/COPD**	9.7 (18/185)	4.0 (1/25)	0.39 (0.49–3.03)	0.366		
**Diabetes mellitus**						
All	23.2 (43/185)	20.0 (5/25)	0.83 (0.29–2.33)	0.717		
Insulin-Dependent	8.7 (16/185)	12.0 (3/25)	1.44 (0.39–5.34)	0.585		
**Renal Failure**	5.9 (10/169)	20.0 (5/25)	3.98 (1.23–12.81)	0.021	2.51 (0.70–8.92)	0.156
**Obesity**	33.7 (57/169)	34.8 (8/23)	1.05 (0.42–2.63)	0.920		
**Extrapulmonary Disease**	26.5 (49/185)	12.0 (3/25)	0.38 (0.11–1.32)	0.128		
**Corticosteroid Treatment**	7.0 (13/185)	8.0 (2/25)	1.15 (0.24–5.43)	0.859		
**Pharmacologic Immunosuppression**	17.8 (33/185)	24.0 (6/25)	1.45 (0.54–3.92)	0.459		
**Solid Organ Transplant**	7.0 (13/185)	12.0 (3/25)	1.80 (0.48–6.83)	0.385		
**Neutrophilia** ^1^	47.8 (75/157)	66.7 (16/24)	2.19 (0.88–5.40)	0.090	2.84 (1.04–7.76)	**0.041**
**Lymphopenia** ^2^	29.9 (47/157)	70.8 (17/24)	5.68 (2.21–14.61)	<0.001	4.50 (1.67–12.11)	**0.003**

Data on survivors and non-survivors are presented as percentages (absolute numbers shown in parentheses), unless otherwise indicated. CI, confidence interval; COPD, chronic obstructive pulmonary disease. ^1^ Neutrophil count ≥ 7.5 × 10^3^cells/microL. ^2^ Lymphocyte count < 1.0 × 10^3^ cells/microL.

**Table 4:** Sensitivity of KOH smear and PCR in respiratory specimens (sensitivity shown as percentage, 95% confidence intervals shown in parentheses).

	All Respiratory Specimens	Sputum	Lower Respiratory Tract Specimens	*p*-Value *
**KOH/Calcofluor White Smear**	46.7	39.1	49.3	0.47
	(36.2–57.4)	(20.5–61.2)	(37.0–61.6)	
	42/90	9/23	33/67	
**PCR**	67.6	45.5	76.9	0.12
	(50.1–81.5)	(18.1–75.4)	(55.9–90.3)	
	25/37	5/11	20/26	

Bronchoalveolar lavage *Aspergillus* galactomannan was positive in 7 of 19 specimens (Sensitivity 36.8% (95% CI, 17.2–61.4)). * Comparison between sputum and lower respiratory tract specimens (tracheal secretions, bronchial washings, bronchoalveolar lavage fluid).

**Table 5:** Sensitivity of diagnostic assays in patients with disease confirmed by culture and/or histopathology with comparison between pulmonary and extrapulmonary disease.

	All	Pulmonary Disease	Extrapulmonary Disease	*p*-Value *
***Blastomyces*** **EIA**	52.1	47.3	68.8	0.13
	(40.7–63.3)	(34.7–60.2)	(44.4–85.8)	
	37/71	26/55	11/16	
***Blastomyces*** **Immunodiffusion**	39.6	36.1	52.6	0.19
	(30.1–49.8)	(26.0–47.7)	(31.7–72.7)	
	36/91	26/72	10/19	
***Blastomyces*** **Complement Fixation**	22.9	17.9	42.9	0.31
	(12.1–39.0)	(7.9–35.6)	(15.8–75.0)	
	8/35	5/28	3/7	
**Urine *Blastomyces* Antigen**	80.8	87.8	54.6	0.03
	(68.1–89.2)	(74.5–94.7)	(28.0–78.7)	
	42/52	36/41	6/11	
**Serum *Blastomyces* Antigen**	64.3	66.7	60.0	1.00
	(38.8–83.7)	(35.4–87.9)	(23.1–88.2)	
	9/14	6/9	3/5	
***Histoplasma* Complement Fixation**	17.9	17.9	None	-
	(7.9–35.6)	(7.9–35.6)		
	5/28	5/28		
***Histoplasma*** **Immunodiffusion**	0/7	0/7	None	-
** Urine *Histoplasma* Antigen**	58.8	58.6	60.0	1.00
	(42.2–73.6)	(40.7–74.5)	(23.1–88.2)	
	20/34	17/29	3/5	

EIA: enzyme immunoassay, BAL: bronchoalveolar lavage. * Comparison between sputum and lower respiratory tract specimens (tracheal secretions, bronchial washings, bronchoalveolar lavage fluid).

**Table 6:** Clinical characteristics of patients with recurrent/relapsed disease.

Age/Sex	Radiographic Findings	Diagnosis	Treatment/Relapse
62 M	Dense left lower lobe consolidation	Culture of bronchial washings	Itraconazole for 7 months Recurrence after 12 months
	Dense right lower lobe consolidation	BAL culture	Itraconazole for 6 months
54 F	Right apical lung cavity and irregular nodules	BAL DNA probe	Itraconazole for 15 months Relapse after 23 months
	Thickening of the cavity wall and cavitating nodules	Urine Antigen (2.73 ng/mL)	Itraconazole for 24 months Relapse after 8 months
	Masslike consolidation in the inferior margins of the cavity	Urine Antigen (2.43 ng/mL)	Itraconazole for 14 months Voriconazole for 25 months
31 M	Right upper lobe mass with rib invasion	Chest wall soft tissue biopsy: granulomatous inflammation	Itraconazole for 9 months Relapse after 9 months
	New T12 vertebral body lesion	Vertebral body biopsy negative for microorganisms, negative culture	Posaconazole for 12 months

## Supplementary Materials

The following are available online at https://www.mdpi.com/article/10.3390/jof7110888/s1, Table S1. Number of cases per year, Table S2. Comparison of characteristics in patients receiving itraconazole or voriconazole as first azole.
