# Supplementary material for: Diagnostic Methods and Risk Factors for Severe Disease and Mortality in Blastomycosis: A Retrospective Cohort Study
Source: J Fungi (Basel). 2021 Oct 20;7(11):888. doi: 10.3390/jof7110888 (PMC8619313; doi:10.3390/jof7110888)
Supplement: Supplementary file 1 [file jof-07-00888-s001.zip › jof-1384285-supplementary.pdf]

Supplementary Table S1. Number of cases per year (Data collected from 1 January 2004 through 31 March 2020)

Year	n
2004	2
2005	9
2006	6
2007	18
2008	10
2009	14
2010	16
2011	19
2012	7
2013	14
2014	13
2015	9
2016	13
2017	19
2018	18
2019	18
2020	5

Supplementary Table S2. Comparison of characteristics in patients receiving itraconazole or voriconazole as first azole.

	**Itraconazole (n=164)**	**Voriconazole (n=19)**	***p*-value**
**Age in years, median (SD)**	50.1 (17.7)	48.9 (18.5)	0.78
**Male Sex**	72.6 (119/164)	57.9 (11/19)	0.18
**Race**			0.34
White	89.5 (136/152)	94.4 (17/18)	
Black	4.0 (6/152)	0	
Asian	5.3 (8/152)	0	
American Indian	1.3 (2/152)	5.6 (1/18)	
Hispanic	2.2 (3/137)	0	1.00
**Central Nervous System Disease**	0.6 (1/164)	26.3 (5/19)	<**0.001**
**Asthma/COPD**	10.4 (17/164)	5.3 (1/19)	0.70
**Diabetes mellitus**			
All	25.0 (41/164)	10.5 (2/19)	0.25
Insulin-Dependent	9.2 (15/164)	10.5 (2/19)	1.00
**Renal Failure**	7.3 (11/151)	5.6 (1/18)	1.00
**Obesity**	35.5 (54/152)	31.3 (5/16)	0.73
**Extrapulmonary involvement**	23.8 (39/164)	42.1 (8/19)	0.10
**Corticosteroid Treatment**	8.5 (14/164)	0	0.37

**Immunosuppressive Therapy**	18.3 (30/164)	21.1 (4/19)	1.00
**Solid Organ Transplant**	6.7 (11/164)	5.3 (1/19)	1.00
**Neutrophilia**	47.9 (68/142)	52.6 (10/19)	0.70
**Lymphopenia**	31.7 (45/142)	42.1 (8/19)	0.36

Data are presented as percentages (absolute numbers shown in parentheses), unless otherwise indicated.

SD, standard deviation; COPD, chronic obstructive pulmonary disease
